# Clinical effect of Zoledronic Acid in the treatment of Senile Osteoporosis

**DOI:** 10.12669/pjms.36.7.1964

**Published:** 2020

**Authors:** Li Kong, Kai Zuo, Long Ma

**Affiliations:** 1 Li Kong, Pharmacy Intravenous Admixture Services, Binzhou People’s Hospital, Shandong 256610, P. R. China; 2 Kai Zuo, Department of Infectious Diseases, Binzhou People’s Hospital, Shandong 256610, P. R. China; 3 Long Ma, Department of Emergency Station, Binzhou People’s Hospital, Shandong 256610, P. R. China

**Keywords:** Zoledronic acid, Senile osteoporosis, Clinical observation

## Abstract

**Objective:**

To investigate the clinical efficacy of zoledronic acid in the treatment of senile osteoporosis.

**Methods:**

One hundred and six cases of senile osteoporosis who visited to our hospital from August 2017 to December 2018 for treatment were selected and randomly divided into a control group and an observation group. The control group was treated with conventional therapy, while the observation group was treated with zoledronic acid in addition to the treatment of the control group. Bone mineral density, pain degree, therapeutic effect and adverse reactions of the two groups were compared.

**Results:**

The total effective rate of the observation group was 96.67%, higher than 80.00% of the control group (P<0.05); the bone mineral density of lumbar vertebrae, femoral neck and Ward’ area in the two groups increased after 6 months of treatment, and the bone mineral density of the observation group increased more than that of the control group (P<0.05); the pain degree of the observation group was lower than that of the control group after 6 months of treatment, and the difference was significant (P<0.05). There was no significant difference in the occurrence of adverse reactions between the two groups (P>0.05).

**Conclusion:**

Zoledronic acid is helpful to alleviate clinical symptoms, reduce the degree of bone pain, and promote the increase of bone mass, and has high safety in the treatment of senile osteoporosis, which is worth promotion.

## INTRODUCTION

Due to the characteristics of dietary structure in China, calcium deficiency is common in the elderly. Moreover, with the increase of age, calcium is losing continuously, which gradually leads to senile osteoporosis.^1^,^2^ The relevant research survey reveals that 70% of the elderly people in China suffer from osteoporosis of varying degrees, and the research value is increasing every year. The elderly patients with osteoporosis have increased bone fragility and are prone to fracture.^3^

The main clinical symptoms of senile osteoporosis are ostealgia, lumbago and leg pain, which have a serious adverse impact on the quality of life of patients.^4^,^5^ Usually, for the treatment of senile osteoporosis, calcium D or bisphosphate is often used clinically. These drugs can effectively alleviate the effects of senile osteoporosis on the body and also can largely ensure the health of the elderly.^6^,^7^ Zoledronic acid is a new generation of bisphosphonates for the treatment of osteoporosis, which has been widely used in the treatment of hypercalcemia caused by tumors such as prostate cancer and multiple myeloma. It has become a hot research topic in recent years. At present, there are related reports on the study of application of zoledronic acid in postmenopausal women and bone metastasis of malignant tumors,^8^-^10^ but the reports on its role in senile osteoporosis are rare. In order to study the therapeutic value of zoledronic acid (Aclasta) in the treatment of senile osteoporosis, the clinical effect of zoledronic acid in the treatment of senile osteoporosis was observed in this study.

## METHODS

One hundred and six elderly osteoporotic fracture patients were selected from August 2017 to December 2018 in our hospital.

### Inclusion criteria

(1) being definitely diagnosed as osteoporosis; (2) all fractures were caused by osteoporosis; (3) being able to effectively tolerate experimental drugs.

### Exclusion criteria

(1) fracture caused by other causes; (2) injury of organs, blood vessels or nerves caused by fracture; (3) complicated with chronic liver and kidney dysfunction, digestive system diseases, thyroid diseases, or malignant tumors. They were randomly divided into an observation group and a control group, 53 cases in each group. There were 28 males and 25 females in the observation group, aged 63-79 (69.46±3.52) years and having a disease course of 1-17 (10.02±6.21) years; there were 16 cases of vertebral fracture, 11 cases of carpal fracture, 23 cases of femoral trochanter fracture and three cases of dorsal pedal fracture. There were 27 males and 26 females in the control group, aged 64-81 (70.02±3.91) years and having a disease course of 2-18 (10.11±6.32) years; there were 14 cases of vertebral fracture, 12 cases of carpal fracture, 21 cases of femoral trochanter fracture, and six cases of dorsal pedal fracture. There was no significant difference in the general data of gender, age, course of disease and type of fracture between the two groups (P>0.05), so the results were comparable. All patients volunteered to participate in the study and signed the informed consent after the approval of the ethics committee (Dated on 15 September 2019).

### Therapeutic method

Both groups were given conventional therapy, i.e., calcium D (Wyeth Pharmaceutical Co., Ltd.; SFDA approval number: H10950029), 600 mg each time, once a day.

The observation group was treated with Novartis Pharma Schweiz AG (SFDA approval number: H201220204): 5 mg of zoledronic acid was mixed in 100 mL of saline for intravenous drip, once a day. The two groups were evaluated after six months of continuous medication. During the treatment, the following nursing measures were taken. The first nursing measure is pain care. Osteoporosis patients often accompany by varying degrees of systemic migratory bone pain. The location, degree, nature and inducing factors of pain in patients were evaluated to help patients remove incentives. When the pain was severe, they were asked to rest in bed and relax. Physical therapy such as infrared ray or ultrashort wave was given to the pain area in proper lying position. If the pain was severe and intolerable, the patients tool analgesics according to the doctor’s advice, and the curative effects of the drugs were observed. The second measure was psychological nursing.

Osteoporosis is a chronic disease. The pain affects the quality of daily life. Patients are prone to negative emotions such as anxiety, tension and fear. Therefore, medical staffs got close to the patients to communicate with them, explain to patients and their families about the professional knowledge of the prevention and control of osteoporosis, and help them understand the risk factors of osteoporosis and osteoporotic fracture. The third measure is drug safety nursing. The pharmacological effect, physicochemical properties and compatibility of drugs are very complex. The influence of many objective factors makes clinicians and nurses unable to consider the stability of drugs, compatibility taboos, repeated administration, and dosage and dilution concentration of drugs comprehensively. In our hospital, pharmaceuticals were dispatched through static dispensing center, and medical orders were strictly checked by pharmaceutical professionals and technicians. Once the above problems arose, the patients could communicate with doctors as soon as possible, so that the problems could be corrected in time and the safety of medication could be improved.

### Observation index

Bone mineral density (BMD) (detected at the lumbar spine, femoral neck and Wards’ area by X-ray bone mineral density detector before treatment and six months after treatment), pain degree (evaluated by visual analogue scale (VAS) method before treatment and six months after treatment, 0 for painless and 10 for severe pain), treatment effect and adverse reactions of the two groups were observed.

### Evaluation criteria for efficacy

Disappearance of clinical symptoms such as pain and significant increase of BMD indicated that the treatment was significant effective. Basic appearance of clinical symptoms and increase of BMD indicated that the treatment was effective. Not satisfying the above standards or aggravation of the disease condition indicated that the treatment was ineffective. Total effective rate=(number of significantly effective cases+number of effective cases)/total number of cases×100%.

### Statistical methods

All data in this study were analyzed by SPSS 22.0. Measurement data were expressed as Mean±SD, and t test was used for inter-group comparison. Counting data were expressed as %, and X^2^ test was used for inter-group comparison. P<0.05 indicated that the difference was statistically significant.

## RESULTS

After six months of treatment, the total effective rate of the observation group was 96.67%, higher than 80.00% of the control group; the difference was statistically significant (P<0.05, Table-I).

**Table-I:** Comparison of curative effects between two groups (%).

Group	Significantly effective	Effective	Ineffective	Total effective rate
Observation group	29(54.7)	21(39.6)	3(5.7)	50(94.3)
Control group	19(35.8)	24(45.3)	10(18.9)	40(81.1)
X^2^	/	/	/	5.543
P	/	/	/	<0.05

Before treatment, there was no significant difference in BMD between the two groups in lumbar vertebrae, femoral neck and Wards’ area (P>0.05); after 6 months of treatment, the BMD of lumbar vertebrae, femoral neck and Wards’ area in the observation group was higher than that in the control group, and the differences were statistically significant (P<0.05, Table-II).

**Table-II:** Comparison of BMD before and after treatment between two groups (g/cm^2^)

Group		Lumbar vertebra	Femoral neck	Wards’ area
Observation group	Before treatment	0.82±0.13	0.61±0.05	0.38±0.07
	After treatment	0.99±0.07^*#^	0.69±0.04^*#^	0.52±0.03^*#^
Control group	Before treatment	0.85±0.12	0.62±0.06	0.39±0.05
	After treatment	0.88±0.11^*^	0.64±0.05^*^	0.46±0.06^*^

* Note: P<0.05 compared to before treatment;

# P<0.05 compared to the control group after treatment.

After six months of treatment, the degree of pain in the two groups was significantly lower than that before treatment (P<0.05); the VAS score in the observation group was lower than that in the control group at the 6^th^ month of treatment (P<0.05), and the difference was statistically significant (P<0.05, Table-III).

**Table-III:** Comparison of pain degree between two groups before and after treatment.

Group	Before treatment	Six months after treatment
Observation group	6.86±1.25	2.10±0.46^*^
Control group	6.73±1.22	3.28±0.63^*^
t	0.475	9.561
P	>0.05	<0.05

* Note: P<0.05 compared to before treatment.

In the treatment period, there were three cases of fever, two cases of nausea and vomiting, and one case of muscle soreness in the observation group; the incidence of adverse reactions was 11.3%. In the control group, there were 2 cases of fever, 2 cases of nausea and vomiting, and one case of muscle soreness; the incidence of adverse reactions was 10.0%. The comparison between the two groups showed that X^2^=0.125, P=0.723>0.05. The adverse reactions of the two groups were mild and were relieved without withdrawal after symptomatic treatment.

## DISCUSSION

In clinic, osteoporosis patients are treated with drugs to increase their BMD and reduce the risk of fracture.^11^ In the past, oral administration of calcium D is usually used to treat senile osteoporosis, which can play a therapeutic role.^12^ However, in clinical application, the therapeutic effect is often limited, the patients’ BMD is not significantly improved after treatment, and the total effective rate is relatively low. It is necessary to use specific drugs for osteoporosis at the same time to improve the clinical efficacy, which was also pointed out by Guo et al.^13^ Therefore, it is of great significance to study the therapeutic effects of calcium agents and specific drugs on senile osteoporosis.

Bisphosphates is featured by strong affinity to bone, fast absorption and good effect. They can maintain effective concentration for a long time in bone and have good clinical acceptance. Previous studies have shown that bisphosphonates are effective in the treatment of osteoporosis and can effectively reduce the risk of fracture.^14^,^15^ In this study, zoledronic acid used by patients in the observation group is one of the bisphosphonate drugs. It is an artificial compound, which can specifically inhibit osteoclast-mediated bone resorption, inhibit the activation of osteoclasts, and have a direct effect on the final formation and activation of osteoclasts to accelerate the apoptosis of such cells and increase BMD.^16^,^17^ In addition, intravenous administration was used in the study, which greatly avoided the adverse reaction of digestive tract caused by oral administration of bisphosphonates and improved the compliance of patients.

The results of this study showed that the total effective rate and BMD of the observation group were higher than those of the control group after treatment, and the pain score was lower than that of the control group, suggesting that zoledronic acid could improve the condition of bone calcium and osteolysis in a certain range, and it could better inhibit bone resorption, alleviate the pain caused by vertebral deformity induced by osteoporosis, and reduce the risk of fracture in such patients compared to the conventional treatment, which was similar to the findings of Sun et al.^18^

Fever is the most common adverse reaction of zoledronic acid. In this study, there were five cases of fever symptom, accounting for 20%, and the highest body temperature was 39 ^o^C, which was similar to the foreign reports.^19^,^20^ The fever disappeared after taking antipyretic analgesics. Moreover two cases had muscle soreness, and one case had flu-like symptom, which were alleviated within 3 days.

## CONCLUSION

Zoledronic acid has a definite clinical effect in the treatment of senile osteoporosis. It can effectively alleviate pain and related clinical symptoms, and the incidence of adverse reactions is low. It is worthy of clinical application. However, due to the limited case number and time, the long-term efficacy of zoledronic acid in elderly patients with osteoporosis remains to be further explored.

### Authors’ Contribution:

**LK:** Study design, data collection, analysis and is responsible for integrity of research.

**LK, KZ & LM:** Manuscript preparation, drafting and revising.

**LK:** Review and final approval of manuscript.
